# Single-cell RNA-seq identified novel genes involved in primordial follicle formation

**DOI:** 10.3389/fendo.2023.1285667

**Published:** 2023-12-11

**Authors:** Hang-Jing Tan, Zi-Heng Deng, Hui Shen, Hong-Wen Deng, Hong-Mei Xiao

**Affiliations:** ^1^ Institute of Reproduction and Stem Cell Engineering, School of Basic Medical Science, Central South University, Changsha, China; ^2^ Center for Reproductive Health, and System Biology, Data Sciences, School of Basic Medical Science, Central South University, Changsha, China; ^3^ Center of Biomedical Informatics and Genomics, Deming Department of Medicine, Tulane University School of Medicine, New Orleans, LA, United States

**Keywords:** primordial follicle formation, single cell RNA sequencing, *in vitro* ovarian culture, premature ovarian insufficiency, whole exome sequencing, whole genome sequencing

## Abstract

**Introduction:**

The number of primordial follicles (PFs) in mammals determines the ovarian reserve, and impairment of primordial follicle formation (PFF) will cause premature ovarian insufficiency (POI).

**Methods:**

By analyzing public single-cell RNA sequencing performed during PFF on mice and human ovaries, we identified novel functional genes and novel ligand-receptor interaction during PFF. Based on immunofluorescence and in vitro ovarian culture, we confirmed mechanisms of genes and ligand-receptor interaction in PFF. We also applied whole exome sequencing (WES) in 93 cases with POI and whole genome sequencing (WGS) in 465 controls. Variants in POI patients were further investigated by in silico analysis and functional verification.

**Results:**

We revealed ANXA7 (annexin A7) and GTF2F1 (general transcription factor IIF subunit 1) in germ cells to be novel potentially genes in promoting PFF. Ligand Mdk (midkine) in germ cells and its receptor Sdc1 (syndecan 1) in granulosa cells are novel interaction crucial for PFF. Based on immunofluorescence, we confirmed significant up-regulation of ANXA7 in PFs compared with germline cysts, and uniform expression of GTF2F1, MDK and SDC1 during PFF, in 25 weeks human fetal ovary. In vitro investigation indicated that Anxa7 and Gtf2f1 are vital for mice PFF by regulating Jak/Stat3 and Jnk signaling pathways, respectively. Ligand-receptor (Mdk-Sdc1) are crucial for PFF by regulating Pi3k-akt signaling pathway. Two heterozygous variants in GTF2F1, and one heterozygous variants in SDC1 were identified in cases, but no variant were identified in controls. The protein level of GTF2F1 or SDC1 in POI cases are significantly lower than that of controls, indicating the pathogenic effects of the two genes on ovarian function were dosage dependent.

**Discussion:**

Our study identified novel genes and novel ligand-receptor interaction during PFF, and further expanding the genetic architecture of POI.

## 1 Introduction

Gametogenesis is a delicate and complex process (1). In female mammals, the primordial germ cells (PGCs) migrate to the gonad and form into cysts by rapidly dividing. Subsequently, oocytes initiate meiosis, cysts are breakdown (CBD), and primordial follicles (PFs) are assembled (2). During the process of CBD and primordial follicle formation (PFF), about two-thirds of oocytes are lost through programmed cell death (PCD), and the remaining oocytes participate in the formation of PFs, which is characterized by a single oocyte surrounded by a layer of pregranulosa cells (pre-GCs) (1). In humans, PFF begins during mid-gestation, while in mice, it begins at embryonic 17.5 days (E17.5) and is completed at postnatal day 3 (P3) (3).

The number of PFs, which is unrenewable, is responsible for subsequent folliculogenesis and oocyte maturation during female adulthood and constitute the ovarian reserve (3). Impairment of PFF will cause premature ovarian insufficiency (POI), which is characterized by menopause before the age of 40 and infertility (4, 5). In some cases, female with mutations in genes responsible for PFF may result in a decrease in ovarian reserve and therefore cause POI (6, 7). In-depth exploration of the molecular mechanisms of oocyte survival/death and oocyte-granulosa cell interactions during PFF can help to elucidate the genetic basis of POI.

Single-cell RNA sequencing (scRNA-seq) allows us to dissect the oocytes and granulosa cells transcriptomes at the single cell level and enables us to explore communication between follicle compartments (3). In this study, we mined publicly available scRNA-seq data on mice and human ovaries during PFF and revealed that ANXA7 (Annexin A7) and GTF2F1 (General transcription factor IIF subunit 1) in germ cells were novel genes regulating PFF. We also identified germ cell ligand midkine (Mdk) and granulosa cell receptor syndecan 1 (Sdc1) as novel interaction in oocytes and pre-GCs. Subsequently, we performed molecular assays to investigate the functional roles of Anxa7 and Gtf2f1 in germ cells, and ligand-receptor (Mdk-Sdc1) interaction during PFF. We also identified novel variants in *GTF2F1* and *SDC1* in POI patients, and explored the pathogenic effects of the POI-associated variants.

## 2 Materials and methods

### 2.1 scRNA-seq data sources and analyses

#### 2.1.1 Data source

The scRNA-seq datasets in the present study were obtained from the Gene Expression Omnibus (GEO) database (https://www.ncbi.nlm.nih.gov/gds) with Accession Number GSE136441 (8) and GSE86146 (9). GSE136441 performed scRNA-seq on C57BL/6 mice ovaries at E16.5, E18.5, P1 and P3. GSE86146 performed scRNA-seq on human ovaries at fetal 16 weeks (16w), 18w, 20w, 23w, 24w and 26w. E16.5-P3 in mice and 16w-26w in humans corresponds to the time of PFF in mice and human, respectively.

#### 2.1.2 Identification of cell types, differentially expressed genes and cell trajectory

The data matrixes were loaded in the Seurat package (version 4.0.2) (10). The Seurat object was created based on 2 filtering parameters of “min. cells = 5” and “low.thresholds = 200”. The exorbitant number of unique genes detected in each cell (i.e., “nFeature_RNA”) was adjusted in each sample to eliminate the empty drop and dying cell. Next, we conducted an integrated analysis for multiple samples. Then, the cells were clustered and visualized using uniform manifold approximation and projection (UMAP) with proper combination of “resolution” and “dims.use”. “FindNeighbors” and “FindClusters” function were conducted to calculate cell clustering.

Cell clusters were annotated according to their markers. Specifically, germ cells were divided into germline cysts and PFs according to the their markers. The DEGs (adjust P-value <0.05 and |logFC| >1) between germline cyst and PFs were determined by the FindMarkers function in Seurat. DEGs in mice and human germ cells were termed Dataset 1 and Dataset 2, respectively.

For cell trajectory analysis, the subgroup of mice germ cells were input into Monocle (version 2.18.0) (11). The new dataset for Monocle object was created based on gene count matrix. Cell trajectories were generated by “reduce Dimension” and “orderCells” function based on pseudotime. “differentialGeneTest” function in Monocle were employed to calculate differentially expressed genes between clusters, which were the ordering genes. Subsequently, differentially expressed genes at key branch points for transition from germline cysts to PFs branch point in the trajectory were calculated by the “BEAM” function. Differentially expressed genes at key branch points for transition from germline cysts to PFs in the mice germ cells trajectory were termed Dataset 3.

#### 2.1.3 Weighted gene co-expression network analysis network construction and hub genes identification

Coexpression network in mice and human germ cells were constructed by WGCNA R package (12). First, we constructed a gene co-expression by R function pickSoftThreshold and calculated the soft thresholding power β. Second, we identified the modules by hierarchical clustering and the dynamic tree cut function. Third, we used correlation between the module eigengene and the phenotype to estimate module-trait associations. Fourth, gene significance (GS) and module membership (MM) were calculated to the modules which most related to traits. Fifth, genes with a GS over 0.2 and an MM over 0.8 in the most trait-relevant module were selected as hub genes, and hub genes in mice and human germ cells were termed Dataset 4 and Dataset 5, respectively.

Common genes in Dataset 1-5 were selected for further functional verification.

#### 2.1.4 Transcriptional regulatory network analysis and transcription factors prediction

The SCENIC (Single Cell Regulatory Network Inference and Clustering) algorithm (13) was applied to assess the regulatory network and discover regulons (TFs and their target genes) in individual cells. Expression matrix generated by Seurat was imported into SCENIC (version 1.2.4). Then, regulons were constructed based on motif dataset (“hg19-tss-centered-10kb-7species.mc9nr.feather”, “hg19-500bp-upstream-7species.mc9nr.feather”, “mm9-500bp-upstream-7species.mc9nr.feather” and “mm9-tss-centered-10kb-7species.mc9nr.feather”). Next, GENIE3 software were used for constructing the co-expressed genes for each TF, followed by Spearman’s correlation between the TF and the potential targets. Subsequently, regulons were generated using the “runSCENIC” function. Finally, AUCell (Area Under the Curve) software were employed to analyze regulon activity, where specific regulons were binarize (“0” present “off” of TFs, and “1” refer to “on”) using a default threshold. The “on” TFs sets of mice germ cells and human germ cells obtained through SCENIC were termed Dataset 6 and Dataset 7, respectively.

Common TFs in Dataset 6 and Dataset 7 were selected for further analysis.

We employed human protein atlas (HPA) database (www.proteinatlas.org/) to analyze common TFs in mice and human germ cells. The TFs with the top one expression in the ovary were selected for further functional analyses.

#### 2.1.5 Cell communication inference

Signal communication among cells were illustrated using Cellchat package (14), which contains ligand-receptor interaction databases for mouse that can analyze the intercellular communication networks from scRNA-seq data annotated as different cell clusters. Cellchat takes the gene expression data of cells as input and created CellChat objects. The interaction dataset of CellChatDB was set as referencing database. Next, the communication probability was computed using a truncated mean of 20% (function computeCommunProb, type = “truncatedMean”, trim = 0.2). After that, the cell-cell communication was inferred and the cell-cell communication network was aggregated with default parameters. The number of interactions was visualized to show the aggregated cell-cell communication network and signaling sent from each cell cluster. Finally, we used netVisual Bubble to display all interactions between oocytes and granulosa cells, and selected novel interactions for subsequent functional analyses.

### 2.2 Functional experiments

#### 2.2.1 Immunofluorescence in human fetal ovary tissue

To validate the expression levels of ANXA7, GTF2F1, MDK and SDC1 in germline cysts and PFs, we obtained human fetal ovary tissue from a fetus after induced abortion at 25 weeks and performed immunofluorescence (IF) on paraffin-embedded tissue. The primary antibodies used in the present study were rabbit polyclonal anti-ANXA7, rabbit polyclonal anti-GTF2F1, rabbit polyclonal anti-MDK, rabbit polyclonal anti-SDC1 and mouse monoclonal anti-DDX4 (**Supplementary Table S1**). In brief, the paraffin sections were subjected to heat-induced antigen retrieval and then incubated overnight with the primary antibody at 4°C. Then samples were incubated with secondary antibodies, either Alexa Fluor 488-conjugated, 555-conjugated anti-mouse or anti-rabbit (1:100; Invitrogen), for 80 min at 37°C. DAPI were used for nuclear DNA staining. The images were acquired on a fluorescence microscope. The signal intensities of the positively stained tissues were analyzed using the mean integrated optical density (mean IOD) with the computer-assisted image system (Image Pro-Plus 6.0, Media Cybernetics, Bethesda, MD, USA).

#### 2.2.2 Animals

All C57BL/6 mice were purchased from Hunan Slake Jingda Experimental Animal Co., Ltd. All mice were exposed to a 12-hour light/dark cycle at a temperature of 21-22 °C and were free to obtain food and water. All animal protocols were approved by the Committee. Adult female mice were mated with male mice, and their vaginal plugs were checked in the following morning. The presence of a vaginal plug was defined as embryonic 0.5 (E0.5). The E17.5 days mice were euthanized and the fetal mouse ovaries were removed for subsequent *in vitro* ovarian culture and interference experiments.

Ovaries were cultured in 1 ml DMEM/F12 mixture (GIBCO, Life Technologies) with 100U/mL Penicillin Streptomycin Liquid (BasalMedia, Shanghai, China) under 37°C, 5% CO_2_. Half of the culture medium was changed every day.

#### 2.2.3 Transfection

To study the functional role of *Anxa7*, *Gtf2f1* and *Mdk* during PFF, Anxa7-shRNA, Gtf2f1-shRNA and Mdk-shRNA (**Supplementary Table S2**) lentiviruses were constructed. The lentivirus expressing a scramble sequence of shRNA was used as a control. 0.3 μL of lentivirus was blown into each mice ovary through an oral pipette. After 3 days of culture, western blot were conducted. After 6 days of culture, immunofluorescence and oocytes counting were conducted. In this experiment, the experimental group and the control group were randomly assigned three ovaries, respectively.

#### 2.2.4 Histological and immunofluorescence analyses in mice ovaries

Every three ovaries were randomly allocated into the control or treated group in one experiment. After 6 days of *in vitro* culture, collected ovaries were fixed in 4% paraformaldehyde (PFA), embedded in paraffin. Ovaries were sliced into 5 μm thick sections, and the sections were stained with Ddx4 antibody and every fifth section was analyzed for the presence of PFs. Number of PFs were counted and multiplied by five to represent each index for one ovary.

Paraffin sections were dewaxed, hydrated, heat-induced for antigen retrieval, and incubated with the primary antibodies (**Supplementary Table S1**) overnight at 4°C. After that, the sections were incubated with the corresponding secondary antibodies at 37 °C for 80 minutes. Finally, DAPI was incubated for nuclear DNA. The images were acquired on a fluorescence microscope. The signal intensities of the Mdk, Sdc1 and Ki67 positively stained tissues were analyzed using the mean integrated optical density (mean IOD) with the computer-assisted image system (Image Pro-Plus 6.0, Media Cybernetics, Bethesda, MD, USA).

#### 2.2.5 Western blot

To explore the effects of Anxa7, Gtf2f1 and Mdk interference on related pathway *in vitro*, western blots were conducted on mice ovaries from the experimental and the control groups. We used RIPA Lysis Buffer (Beyotime Biotechnology, Shanghai, China) with protease inhibitor Phenylmethylsulfonyl fluoride (PMSF) to extract total protein from cells. Next, 10% sodium dodecyl sulphate polyacrylamide gel electrophoresis were used to resolve 20 μg of the protein, and the bands were then electro-blotted onto polyvinylidene difuoride membranes (Millipore, Shanghai, China). Subsequently, the membranes were blocked with 5% skim milk for 1h and then incubated overnight at 4°C with the following primary antibodies. The primary antibodies used in the study were presented in **Supplementary Table S1**. After the incubation of the first antibody, the membranes were washed three times with Tris Buffered Saline Tween-20 (TBST). Following this, the membranes were incubated with HRP-conjugated goat anti-rabbit IgG (1:5 000, Proteintech, Wuhan, China) or goat anti-mouse IgG (1:5 000, Proteintech, Wuhan, China). We then used enhanced chemiluminescence reagent to visualize signals according to the manufacturer’s protocol. The band intensity was quantified using Gapdh as internal control and measured with computer-assisted image system (Image Pro-Plus 6.0, Media Cybernetics, Bethesda, MD, USA).

### 2.3 Genetic testing in POI patients and healthy controls

Ninety-three POI patients were recruited from Changsha Jiangwan Obstetrics and Gynecology Hospital, Xiangya Second Hospital, and Hunan Provincial Maternal and Child Health Hospital. The recruited POI patients must meet the following two conditions: (1) primary or secondary amenorrhea before age of 40; (2) serum follicle-stimulating hormone (FSH) > 25 IU/L on two occasions >4 weeks apart. Patients with chromosomal abnormalities, FMR1 variants, pelvic surgery, endometriosis, ovarian infection, radiotherapy/chemotherapy, and endocrine autoimmune diseases were excluded. Blood samples (2–3 mL) were obtained using EDTA anticoagulation tubes. Genomic DNA samples were extracted and stored at −80°C. We sequenced the exomes of the 93 POI patients using Agilent SureSelect Whole Exome capture and Illumina sequencing technology (SierraVasr Bio-Medical, Shanghai) following the manufacturer’s protocol. We also performed Sanger sequencing to validate the identified *GTF2F1* or *SDC1* mutations (see the **Supplementary Appendix**).

A cohort of 465 women aged 45–65 years in the menopause stage (no menstruation/amenorrhea for ≥3 months) were used as healthy controls. This cohort was recruited from The Third Affiliated Hospital of Southern Medical University in 2017 and has been previously tested by whole genome sequencing analyses (15).

### 2.4 *In silico* and functional analyses of identified mutations in POI patients and controls

To assess the potential functional impact of the identified mutations, we used 11 in silico tools, including MutationTaster (http://www.mutationtaster.org/) (16), Combined Annotation Dependent Depletion (CADD, https://cadd.gs.washington.edu/snv) (17), deleterious annotation of genetic variants using neural networks (DANN, https://cbcl.ics.uci.edu/public_data/DANN/) (18), FATHMM_MKL (http://fathmm.biocompute.org.uk/fathmmMKL.htm) (19), fitness consequence (fitCons, http://compgen.cshl.edu/fitCons2/) (20), Genomic Evolutionary Rate Profiling_plus (GERP++, http://mendel.stanford.edu/SidowLab/downloads/gerp/index.html) (21), phyloP (http://ionreporter.thermofisher.com/) (20), PhastCons (http://varianttools.sourceforge.net/Annotation/PhastCons) (20), SIFT (http://sift.bii.a-star.edu.sg/) (22), Polyphen2 (http://genetics.bwh.harvard.edu/pph2) (23) and GenoCanyon (GitHub - GeneticResources/AnnoPred) (24). We also used the amino acid sequences of different species to analyze the evolutionary conservation of the identified mutations. Sequences were obtained from the UCSC (University of California Santa Cruz) Genome Browser. The structure of the GTF2F1 and SDC1 protein was modeled by AlphaFold (AlphaFold Protein Structure Database), and we used PyMol (http://www.pymol.org) for structure visualization.

To assess the effects on protein levels of the identified *GTF2F1* or *SDC1* mutations, we compared GTF2F1 or SDC1 levels in peripheral blood plasma using enzyme linked immunosorbent assay (ELISA) between POI patients with *GTF2F1* or *SDC1* mutation and three age-matched healthy women (see the **Supplementary Appendix**).

### 2.5 Statistical analysis

All experiments were performed in triplicate. The data were expressed as means ± standard errors and analyzed by GraphPad Prism 9 (La Jolla, CA, USA). Differences between two groups were evaluated with Student’s t tests. P values of less than 0.05 were considered statistically significant.

## 3 Results

### 3.1 Study design

**Figure 1:**
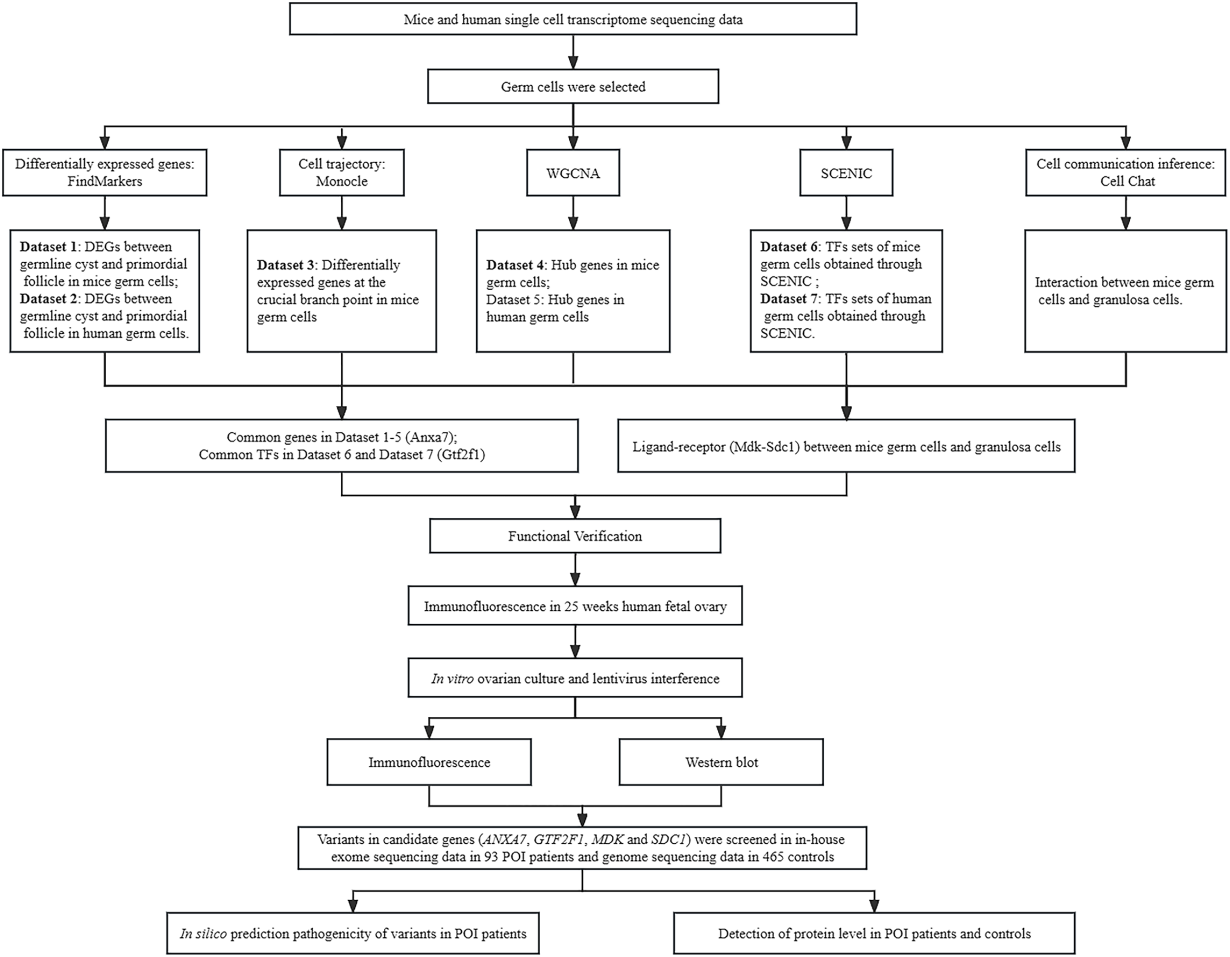
Study design. DEGs, Differentially Expressed Genes; WGCNA, Weighted gene co-expression network analysis; SCENIC, Single Cell Regulatory Network Inference and Clustering.

The study design is shown in **Figure 1**. In our current research, single cell RNA-seq on mice and human developing ovaries were analyzed by a series of bioinformatics methods. We selected common key genes in mice and human germ cells, and oocyte-granulosa cell interactions for further verification.

### 3.2 Gene expression signatures of the mice and human germ cells from the germline cysts to the primordial follicle

**Figure 2:**
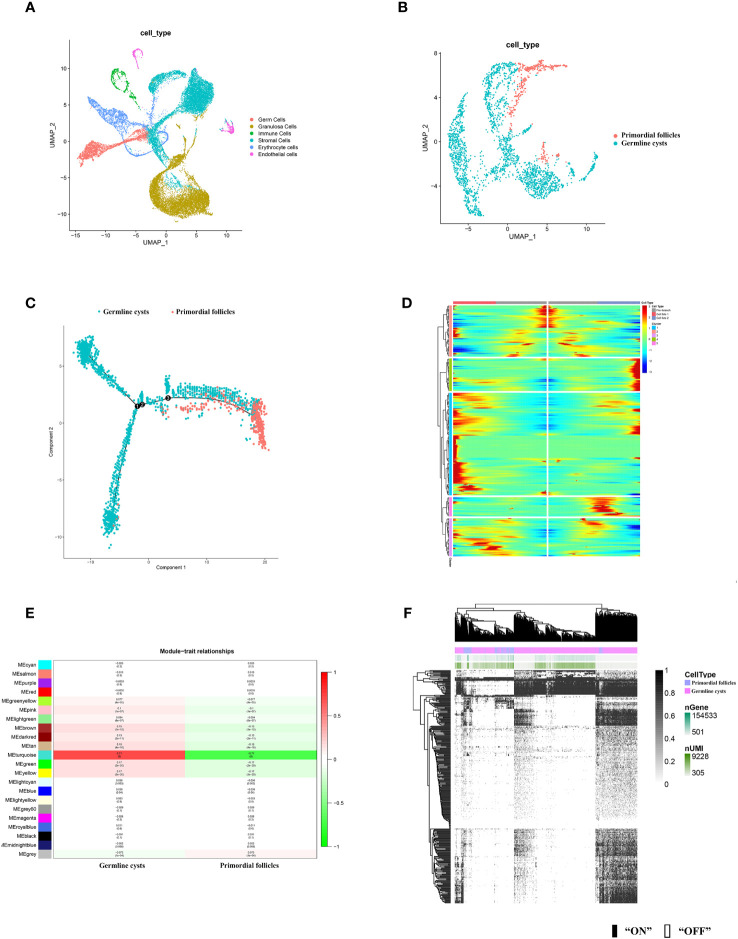
scRNA-seq identified mice ovarian cell types and molecular characterization of the mice germ cell subsets during PFF. **(A)** UMAP plot of 6 ovarian cell types. **(B)** UMAP plot of mice germ cells in germline cysts and PFs. **(C)** Single-cell trajectories of mice germ cell. **(D)** Heatmap representing the expression dynamics of five gene sets with increased expression or reduced at the germline cysts and follicle stage. Heatmap showed genes with “qval < 1 × 10^−4^”. **(E)** WGCNA showed module-trait relationships of genes in germ cells of germline cysts and PFs. Each row represents a module eigengene, and each column represents a trait. Genes clusters (modules) were displayed in different colors. In each module, red and green blocks represents positive and negative correlation with the trait, respectively. The higher the absolute value of the number in the module, the more relevant the module is to the trait. **(F)** Heatmap of germ cells regulon activity analyzed by SCENIC with default thresholds for binarization. “On” indicates active regulons; “Off” indicates inactive regulons. The top rows represent the transcriptome signature of selected germ cells. Each row in the image represents a transcription factor.

**Figure 3:**
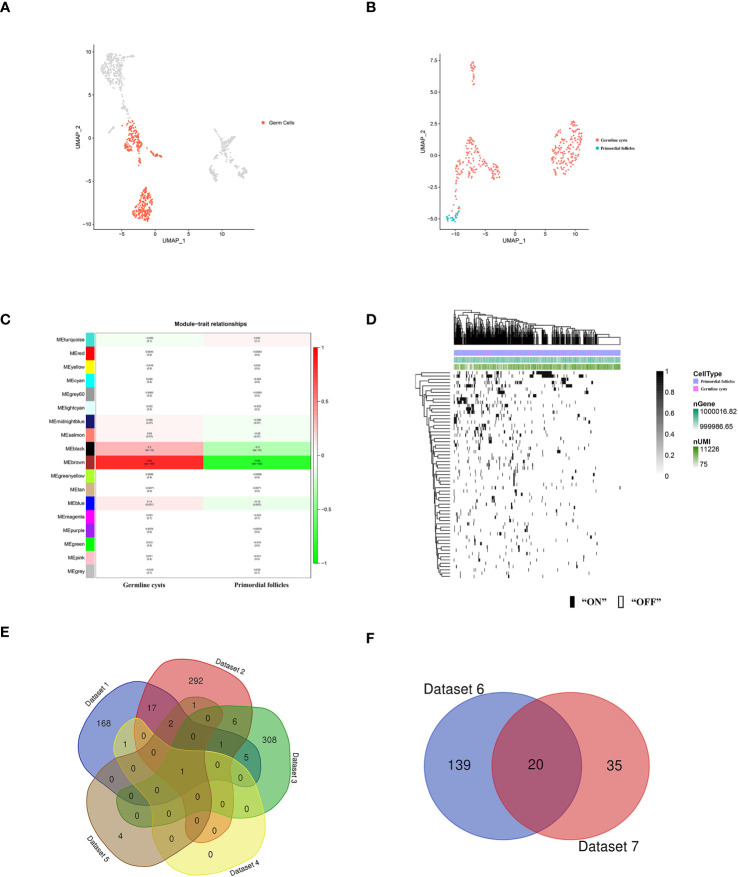
scRNA-seq identified human germ cells and molecular characterization of the human germ cell subsets during PFF. **(A)** UMAP plot of human germ cell types. **(B)** UMAP plot of human germ cells in germline cysts and PFs. **(C)** WGCNA showed module-trait relationships of genes in human germ cells of germline cysts and PFs. Each row represents a module eigengene, and each column represents a trait. Genes clusters (modules) were displayed in different colors. In each module, red and green blocks represents positive and negative correlation with the trait, respectively. The higher the absolute value of the number in the module, the more relevant the module is to the trait. **(D)** Heatmap of human germ cells regulon activity analyzed by SCENIC with default thresholds for binarization. “On” indicates active regulons; “Off” indicates inactive regulons. The top rows represent the transcriptome signature of selected germ cells. Each row in the image represents a transcription factor. **(E)** Venn diagram showing common genes in Dataset 1-5. **(F)** Venn diagram showing common genes in Dataset 6 and Dataset 7.

According to the distinct marker gene (**Supplementary Figure S1**), six cell types in mice ovary were identified, including germ cells, granulosa cells, immune cells, stromal cells, erythrocytes, endothelial cells (**Figure 2A**). Human germ cells were also identified and extracted according to the distinct marker gene (**Figure 3A**).

To further explore the changes of germ cells during PFF, we extracted the data from mice and human germ cells and divided the germ cell population into germline cysts and PFs according to the markers (mice: **Figure 2B**; **Supplementary Figures S2**, **S3**, human: **Figure 3B**; **Supplementary Figure S4**). In mice, 237 DEGs were identified, including 152 up-regulated DEGs and 85 down-regulated DEGs, in PFs compared to germ cell cysts (**Supplementary Table S3**) (Dataset 1). In human, 366 DEGs were sifted out, including 215 up-regulated DEGs and 151 down-regulated DEGs in PFs compared to germline cysts (**Supplementary Table S7**) (Dataset 2).

In addition, to clarify the developmental trajectory of germ cells during PFF, mice germ cells were ordered along pseudotime trajectories (**Figure 2C**). Three branches were identified and the PFF began from the third branch node. Differentially expressed genes at branch point three can be divided into five clusters (**Figure 2D**), with a total of 405 genes (**Supplementary Table S4**) (Dataset 3). These five clusters, with distinct expression patterns, were likely involved in the commitment of germ cells within cysts into follicles. The heatmap showed the dynamics of gene expression in the germ cells at the cysts and follicle stages. Genes in cluster 1 seemed to function mainly at pre-follicle formation stages and was significantly decreased in germ cells within follicles. Cluster 2 includes genes with steady high expression in germ cells within follicles. Cluster 3 comprised genes specifically up-regulated in germ cells in cysts. Genes in cluster 4 seemed to function mainly at early follicle formation stages and was significantly decreased for germ cells within follicles. Cluster 5 may comprised genes responsible for CBD and decreased in germ cells within follicles.

In mice, WGCNA identified 22 modules and showed that the turquoise module was closely related to PFF (**Figure 2E**). Using a GS over 0.2 and an MM over 0.8 as cut-off criteria, two genes (*Cd164l2* and *Anxa7*) were identified as hub genes of the turquoise module (**Supplementary Table S5**) (Dataset 4). In human, WGCNA identified 18 modules and showed module brown was closely related to PFF (**Figure 3C**). Using a GS over 0.2 and an MM over 0.8 as cut-off criteria, nine genes (*ANXA7*, *CSF1R*, *DGKB*, *FAM167A-AS1*, *NLRP5*, *NTN1*, *PADI6*, *SARDH*, *ZAR1*) were sifted out as hub genes (**Supplementary Table S8**) (Dataset 5).

SCENIC algorithm identified 168 TFs and 63 TFs in mice germ cells (**Figure 2F**) (**Supplementary Table S6**) (Dataset 6) and human germ cells (**Figure 3D**) (**Supplementary Table S9**) (Dataset 7), respectively.

Common gene in Dataset 1-5, ANXA7, was selected for further functional verification (**Figure 3E**). In addition, there were 20 common TFs identified in mice and human germ cells including *SIN3A*, *SP1*, *YY1*, *FOS*, *JUNB*, *ATF2*, *MSX1*, *NFIC*, *JUN*, *TEAD1*, *LHX9*, *FIGLA*, *POU5F1*, *GATA4*, *EGR1*, *FOXJ2*, *GTF2F1*, *HDAC6*, *GATA6* and *THRA* (**Figure 3F**).

Among these common TFs, *YY1 (* 25), *FIGLA* (26), *SP1* (27), *HDAC6* (28), *POU5F1* (28), *JUN* (29), *MSX1* (30), *LHX9* (31), *JUNB* (32), *FOS* (33), *GATA6* (34), *GATA4* (34) and *EGR1* (35) have been reported to be involved in folliculogenesis. Among the remaining seven common TFs, the HPA database predicts that GTF2F1 expressed highest in the human ovary compared to the other tissues (**Supplementary Figure S5**). Therefore, we also chose GTF2F1 for further functional study. Other genes, such as *ATF2* and *SIN3A*, are not the highest in human ovary compared to other tissues, but they may also function during PFF, and needs exploration in future study.

### 3.3 Communication between germ cells and granulosa cells during PFF

**Figure 4:**
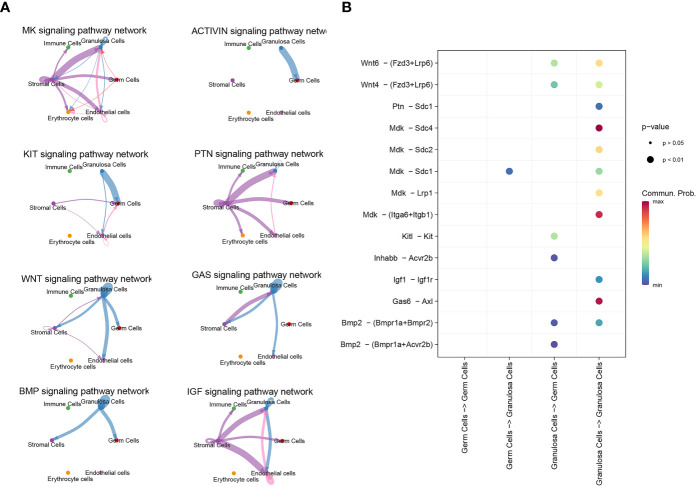
scRNA-seq identified signal communication between oocyte and granulosa cells. **(A)** Signal pathways between oocytes and granulosa cells, and signal pathways between granulosa cells themselves. **(B)** The communicating molecules between oocytes and granulosa cells.

CellChat detected a number of significant signaling pathway among the 6 cell groups. There are only five signal pathways, including MK, WNT, KIT, BMP and Activin signaling pathway, existing between germ cells and granulosa cells (**Figure 4A**). Specific molecules that communicate between germ cells and granulosa cells were shown in **Figure 4B**. The role of ligand Mdk in oocytes and its receptor Sdc1 in granulosa cells was the only unclear ligand-recptor pair sifted out by CellChat during PFF.

### 3.4 The expression of ANXA7, GTF2F1, MDK and SDC1 in human fetal ovary

**Figure 5:**
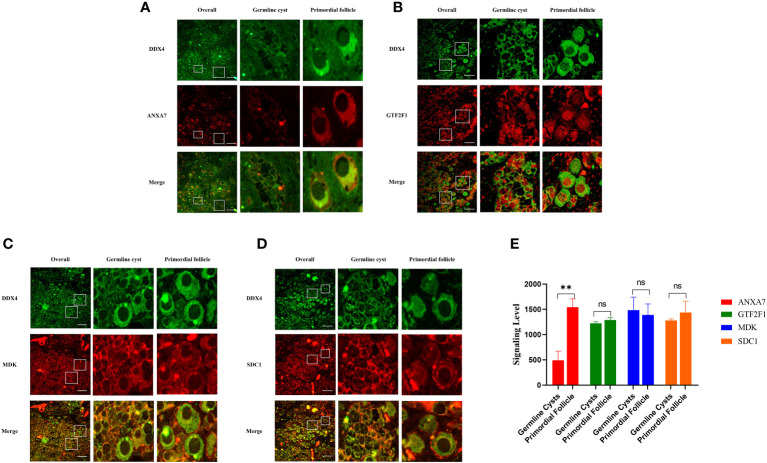
Representative images of the ANXA7, GTF2F1, MDK and SDC1 staining of human ovarian sections at 25 weeks of gestation. Panel **(A-D)** shows immunofluorescence analysis of the expression of ANXA7, GTF2F1, MDK and SDC1 in germline cysts and PFs, respectively. Scale bars: 100 μm. Panel **(E)** shows mean IOD of the expression of ANXA7, GTF2F1, MDK and SDC1 in germline cysts and PFs. All experiments were repeated at least three times. Data were presented as mean ± SD (**, P<0.01. ns; no significance).

To investigate whether ANXA7, GTF2F1, MDK and SDC1 were involved in PFF, we first measured the expression of these proteins in human perinatal ovaries. The results indicated that expression level of ANXA7 was increased in PFs compared to germline cysts, while the expression levels of GTF2F1, MDK and SDC1 were uniform during PFF (**Figure 5**). These results were consistent with the bioinformatics analysis.

### 3.5 Anxa7, Gtf2f1 and Mdk-Sdc1 regulates primordial follicle formation in mice

ANXA7 acts as a critical regulator of JAK/STAT3 pathway (36), while JAK/STAT3 pathway contributes to PFF (37). GTF2F1 can interact with JNK in yeast (38), while JNK signaling promote PFF by regulating E-cadherin (29). However, the role of ANXA7 and GTF2F1 in PFF and POI were currently unclear, as well as their potential mechanism.

**Figure 6:**
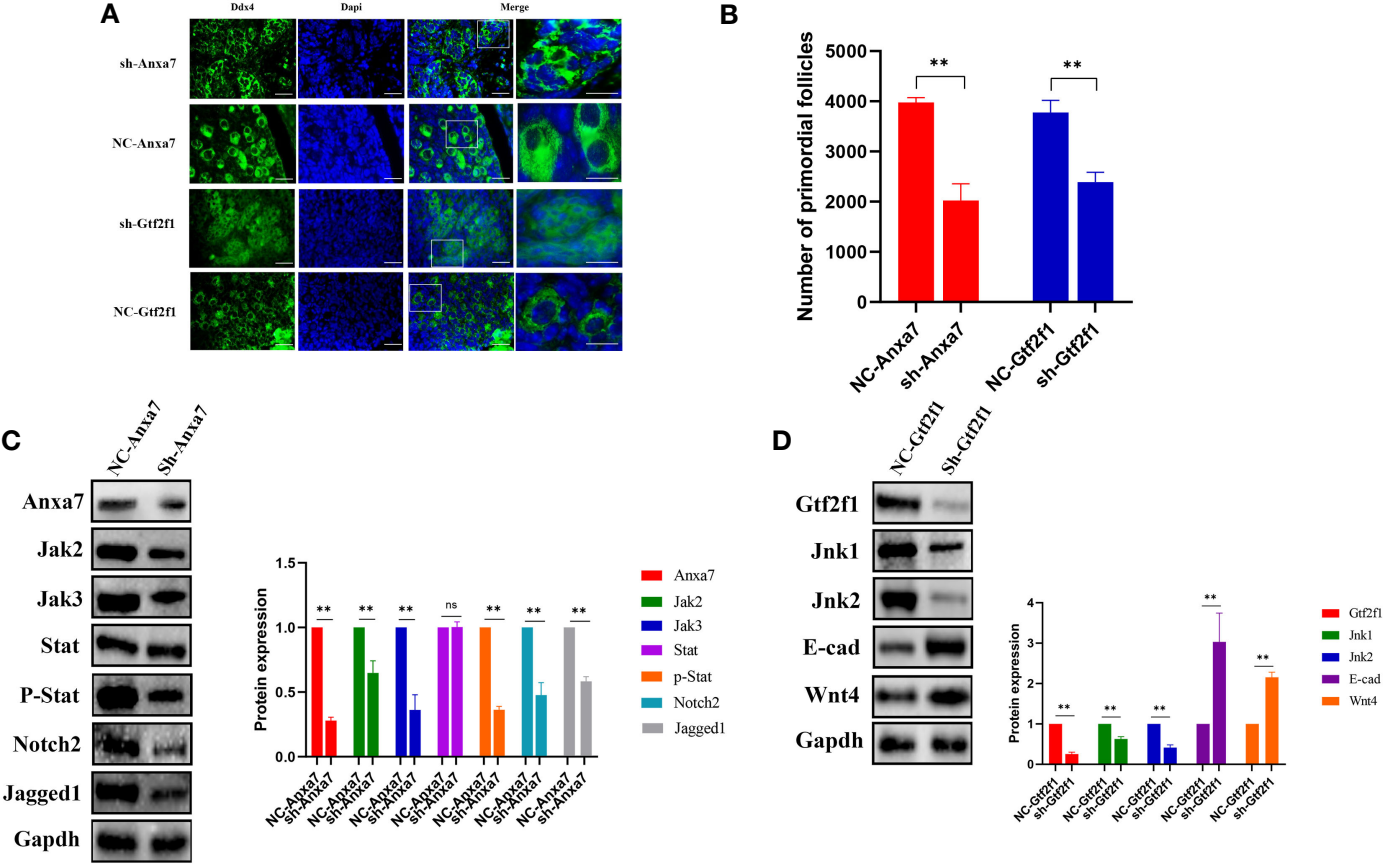
Effects of Anxa7 or Gtf2f1 suppression on primordial follicle formation. **(A)** Ovaries at E17.5 were cultured with sh-Anxa7 or sh-Gtf2f1 *in vitro*. After 6 days of culture, ovarian sections were immunolabeled with Ddx4 (green) and the nuclear marker dapi (blue). NC: negative control. Scale bars: 20 μm. **(B)** The number of PFs was quantified (**, P<0.01). **(C)** After three days of culture, Anxa7, Jak2, Jak3, Stat3, phospho-Stat3, Notch2, and Jagged 1 expression levels in sh-Anxa7 or negative control (NC)-treated ovaries were analyzed by western blot. Gapdh served as a loading control. **(D)** After three days of culture, Gtf2f1, Jnk1, Jnk2, E-cad and Wnt4 levels in sh-Gtf2f1 or negative control (NC)-treated ovaries were analyzed by western blot. Gapdh served as a loading control. The relative protein levels were calculated from the band intensities. All experiments were repeated at least three times. Data were presented as mean ± SD (**, P<0.01, ns, no significance).

In mice, PFF begins at E17.5 and is completed within the first 3 days after birth. We can culture the mice ovaries of E17.5 *in vitro* for 6 days to observe the formation of PFs under different treatment. To explore the role of Anxa7 and Gtf2f1 in PFF, E17.5 fetal ovaries were collected and cultured *in vitro*. Anxa7-shRNA and Gtf2f1-shRNA lentiviruses were used to knockdown Anxa7 and Gtf2f1 expression in the fetal mice ovary. After 3 days of transfection, protein levels of Anxa7 and Gtf2f1 were efficiently downregulated (**Figures 6C, D**). After 6 days of transfection, compared with controls, ovaries transfected with Anxa7-shRNA or Gtf2f1-shRNA lentiviruses have significantly less primordial follicles (**Figures 6A, B**).

To further elucidate the molecular mechanisms of Anxa7 and Gtf2f1 on PFF, we evaluated the gene expression and activities of Jak/Stat3 and Jnk signaling pathways, in E17.5 ovaries transfected with Anxa7-shRNA or Gtf2f1-shRNA lentiviruses, respectively. The results revealed that knockdown of Anxa7 decreased the expression levels of Jak1, Jak2 and the ratio of p‐Stat3/Stat3 (**Figure 6C**), as well as Jagged1 and Notch2, two genes interacting with the Jak/Stat3 pathway (37). Similarly, knockdown of Gtf2f1 decreased the expression level of Jnk and increased the expression of E-cadherin and Wnt4 (**Figure 6D**).

**Figure 7:**
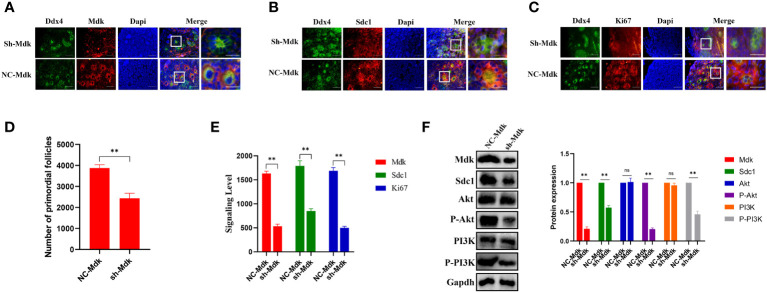
Effects of Mdk suppression on primordial follicle formation and granulosa cells. **(A)** Ovaries at E17.5 were cultured with sh-Mdk or negative control (NC) *in vitro*. After 6 days of culture, ovarian sections were immunolabeled with Ddx4 (green), Mdk (red) and the nuclear marker dapi (blue). Scale bars: 20 μm. **(B)** Ovaries at E17.5 were cultured with sh-Mdk or negative control (NC) *in vitro*. After 6 days of culture, ovarian sections were immunolabeled with Ddx4 (green), Sdc1 (red) and the nuclear marker dapi (blue). Scale bars: 20 μm. **(C)** Ovaries at E17.5 were cultured with sh-Mdk or negative control (NC) *in vitro*. After 6 days of culture, ovarian sections were immunolabeled with Ddx4 (green), Ki67 (red) and the nuclear marker dapi (blue). Scale bars: 20 μm. **(D)** After 6 days of culture, the number of PFs was quantified. (**, P<0.01, ns: no significance). **(E)** Mean IOD of the expression of Mdk, Sdc1 and Ki67 in sh-Mdk-treated and negative control (NC)-treated ovaries. **(F)** After three days of culture, Mdk, Sdc1, Akt, phospho-Akt, PI3K, and phospho-PI3K levels in sh-Mdk or negative control (NC)-treated ovaries were analyzed by western blot. Gapdh served as a loading control. The relative protein levels were calculated from the band intensities. All experiments were repeated at least three times. Data were presented as mean ± SD (**, P<0.01. ns, no significance).

SDC1, receptor of MDK, is involved in mediating cell binding, cell signaling and cytoskeletal organization (39). Upregulation of SDC1 significantly over-activated PI3K/AKT signaling, thus alleviated drug resistance in hepatic cancer (40). PI3K/AKT signaling can affect the proliferation of pre-GCs, which is widely accepted to be involved in cyst breakdown and PFF (41). However, the role of MDK-SDC1 in the PFF and POI remains unclear. To determine whether ligand-receptor (Mdk-Sdc1) interaction plays vital roles in PFF, E17.5 ovaries were cultured *in vitro* with Mdk-shRNA lentiviruses. After 3 days of transfection, Mdk protein levels were significantly downregulated (**Figure 7F**). After 6 days of transfection, compared with controls, ovaries transfected with Mdk-shRNA lentiviruses have significantly less primordial follicles (**Figure 7D**). IF revealed that Mdk protein level in oocytes and Sdc1 protein level in granulosa cells, were downregulated (**Figures 7A, B, E**). In addition, the expression of Ki67, a marker of proliferating cells, was significantly downregulated (**Figures 7C, E**). Further western blot study revealed that downregulation of Mdk reduced proliferation of pre-GCs by inhibiting Pi3k/akt signaling (**Figure 7F**), and thus impaired PFF.

### 3.6 Identification of *GTF2F1* or *SDC1* mutations associated with POI

To explore whether genetic mutations in *ANXA7*, *GTF2F1*, *MDK* and *SDC1* genes were associated with POI, we performed sequence analyses in 93 POI patients and 465 health controls. Using genetic frequencies less than 5% in gnomAD database (gnomad-sg.org) as screening criteria, no variant of *ANXA7* and *MDK* was identified in either the POI patients or the control group. Two novel coding variants of *GTF2F1* (NM_002096. c.943A>G, p.Lys315Glu; c.595C>T, p.Arg199Cys) and one novel coding variant of *SDC1* (NM_001006946. c.461A>G, p.His154Arg) were detected in three POI patients (**Supplementary Table S10**), but not in controls.

### 3.7 The impact of *GTF2F1* and *SDC1* mutation

**Figure 8:**
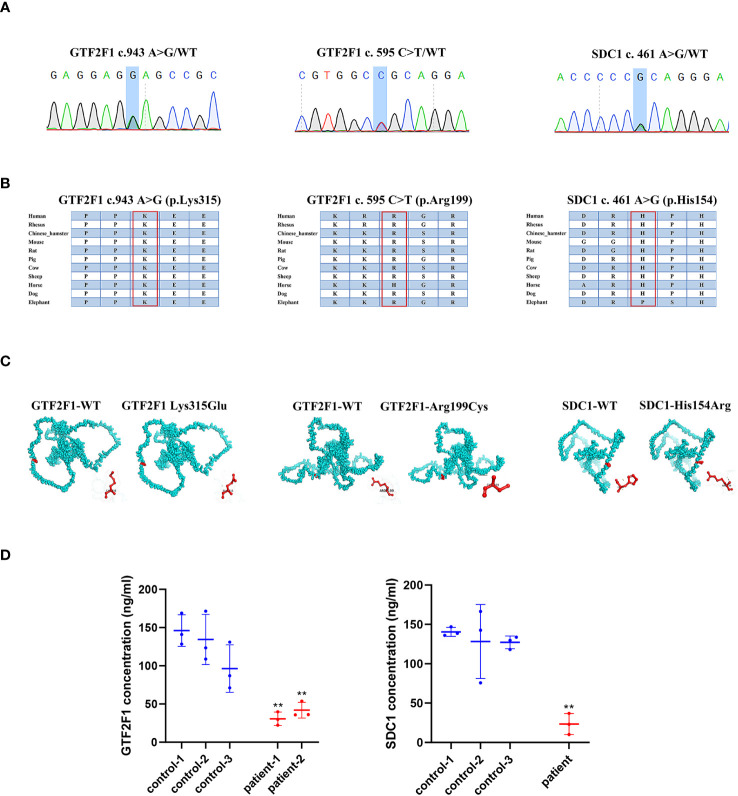
The impact of *GTF2F1* and *SDC1* mutation. **(A)** Chromatograms of the three heterozygous variants. **(B)** The mutant amino acids were highly conserved in different species. **(C)** Modeling of the GTF2F1 and SDC1 wide type and mutant protein. **(D)** Protein level of GTF2F1 and SDC1 in POI patients and controls. Patient -1 represents a patient with c.943A>G heterozygous mutation. Patient-2 represent patient with c.595C>T heterozygous mutation. (**, P<0.01).

The POI patients with *GTF2F1* or *SDC1* mutation (**Figure 8A**) suffered secondary amenorrhea before 40 years old. The clinical characteristics of the heterozygote are shown in **Supplementary Table S10**. The potential pathogenicity of GTF2F1 Lys315Glu and Arg199Cys, and SDC1 His154Arg was analyzed by different *in silico* tools (**Supplementary Table S11**). Eight and three different prediction software predicted these novel variants in *GTF2F1* and *SDC1* to be deleterious, respectively. We also compared the protein sequences among different species (Human, Mouse, Rat, Pig, Cow, Sheep, Dog, Elephant) and found Lys315 and Arg199 in GTF2F1, and His154 in SDC1 were 100% conserved in these species (**Figure 8B**). These results implied that Lys315 and Arg199 in GTF2F1, and His154 in SDC1were located in a functional domain.

The variant (NM_002096, c.943A>G, p.Lys315Glu) caused the 315th amino acid of the GTF2F1 protein to change from lysine to glutamic acid (**Figure 8C**). The differences in amino acid properties can result in disturbance of the protein function. For example, the mutant residue is smaller than the wild-type residue, which might lead to loss of interactions. In addition, the wild-type residue charge was positive, but the mutant residue charge is neutral, which may cause loss of interactions with other molecules or residues (42). Similar results were also found for the variant (NM_002096, c.595C>T, p.Arg199Cys), where the mutant residue is smaller and causes the loss of positive charge at this residue position (**Figure 8C**). In addition, the mutation introduces a more hydrophobic residue at this position, which can result in loss of hydrogen bonds and/or disturb correct folding (42).

The variant (NM_001006946. c.461A>G, p.His154Arg) caused the 154th amino acid of the SDC1 protein to change from histidine to arginine (**Figure 8C**). The mutant residue is bigger, which might lead to bumps. The wild-type residue charge was neutral, the mutant residue charge is positive. The mutation introduces a charge, which can cause repulsion of ligands or other residues with the same charge (42).

We compared GTF2F1 and SDC1 levels in plasma of POI patients with *GTF2F1* or *SDC1* mutation and three age-matched healthy controls and found that the mutants in *GTF2F1* (p.Lys315Glu and p.Arg199Cys) and *SDC1* (p.His154Arg) lead to decreased GTF2F1 and SDC1 levels, respectively (**Figure 8D**).

## 4 Discussion

In mammals, PFF is a crucial process to regulate ovarian reserve and greatly affect female fertility. Female with mutations in genes responsible for PFF may result in a decrease in ovarian reserve and therefore cause POI. In this context, combined with *in silico* and *in vitro* study, we identified *Anxa7*, *Gtf2f1*, and ligand-receptor (*Mdk*-*Sdc1*) as potential drivers in PFF. Also, through mutation screening in POI patients and healthy controls, we identified *GTF2F1* and *SDC1* might be novel genes leading to POI.

Germ cells are crucial during PFF, we therefore focused on germ cell types. Previous studies revealed that *Lhx8*, *Sohlh1*, *Nobox* and *Ooep* were highly expressed in PFs, while *Meioc*, *M1ap*, *Hspb11* and *Top2a* were highly expressed in germline cysts (1). Based on the known stage-specific genes, the transcriptome changes in germ cells at germline cysts and PFs were revealed. We also discovered novel genes, including *Anxa7* and *Gtf2f1* expressed by germ cells during PFF. These genes are therefore the focus of our further studies.

Annexin A7, a member of calcium ion dependent phospholipid binding annexin family, has a series properties, such as phospholipid vesicle fusion and exocytosis (36). Previous studies related to ANXA7 focused more on carcinoma, including hepatocarcinoma, prostate carcinoma and breast cancer. Mechanically, ANXA7 acts as a critical regulator of JAK/STAT3 pathway (36), while JAK/STAT3 pathway contributes to PFF (37). However, the role of ANXA7 and annexin in PFF has not been reported. Our results demonstrated that knockdown of Anxa7 leads to a significant downregulation of Jak2, Jak3 and phosphorylation of Stat3, which impair PFF. Therefore, we emphasized the role of Jak/Stat3 signaling in PFF process, and we also supposed that Anxa7, might be involved in PFF.

General transcription factor Gtf2f1 is involved in several functions, such as interacting with JNK in yeast (38), while JNK signaling promote PFF by regulating E-cadherin (29). However, the role of GTF2F1 in PFF and POI were currently unclear, as well as their potential mechanism. Our results demonstrated that knockdown of Gtf2f1 leads to a significant downregulation of Jnk signaling and upregulation of E-cadherin and Wnt4, which impair PFF. Our study also emphasized the role of the JNK signaling in PFF, and speculated that molecules which regulate the JNK signaling, E-cadherin, or WNT4 may participate in the PFF process. In addition, previous studies pay more attention to specific transcription factors, such as SOHLH1 (spermatogenesis and oogenesis specific basic helix-loop-helix 1), NOBOX (oogenesis homeobox), FIGLA (folliculogenesis specific bHLH transcription factor), during folliculogenesis (26). However, we highlighted that general transcription factors may also play an important role in PFF.

PFF also requires interaction between germ cells and surrounding granulosa cells. In accordance with previous studies, we showed that several signaling pathways, such as BMP (4), WNT4 (29) and Kit signals (4), were involved in PFF. In addition, we revealed that MK signaling may be a novel pathway activated during PFF. SDC1, receptor of MDK, is involved in mediating cell binding, cell signaling and cytoskeletal organization (39). Upregulation of SDC1 significantly over-activated PI3K/AKT signaling, thus alleviated drug resistance in hepatic cancer (40). PI3K/AKT signaling can affect the proliferation of pre-GCs, which is widely accepted to be involved in cyst breakdown and PFF (41). However, the role of MDK-SDC1 in the PFF and POI remains unclear. Our functional studies better delineated the communication between germ and granulosa cells. Knockdown of Mdk in germ cells reduced Sdc1 expression in pre-GCs, and reduced proliferation of pre-GCs by PI3K/AKT signaling, thus impaired PFF. Previous studies implicated that dysregulation of MK signaling is involved in a variety of inflammatory diseases and cancers (43). However, our studies proposed that MK signaling may also be involved in folliculogenesis, such as PFF.

Genetic mutations account for 20% to 25% of POI cases (44). In most cases, impairment of and PFF may result in POI (6, 7). Our results provided a good resource for screening POI candidate genes when combined with pedigree or population-based studies. Through screening our in-house databases, we identified *GTF2F1* and *SDC1* might be novel genes causing POI by haploinsufficiency effect. However, we did not identify mutation in *ANXA7* and *MDK* genes, which might result from small sample size of POI patients.

In conclusion, in addition to transcriptional dynamics in germ cells and communication network in germ cells and granulosa cells during PFF, our study further identified the novel genes (*Anxa7* and *Gtf2f1*) and novel ligand-receptor (*Mdk*-*Sdk1*) promoting PFF, and clarified pathogenic variants in *GTF2F1* and *SDC1* in POI patients. These findings expanded the genetic spectrum of POI and highlighted the essential role of PFF genes in maintenance of ovarian function.

## Data availability statement

The datasets presented in this study can be found in online repositories. The names of the repository/repositories and accession number(s) can be found below: https://www.ncbi.nlm.nih.gov/geo/, GSE136441 https://www.ncbi.nlm.nih.gov/geo/, GSE86146.

## Ethics statement

This study was approved by the Ethics Committees of School of basic medicine, Central South University (2022-KT110). The studies were conducted in accordance with the local legislation and institutional requirements. Written informed consent for participation in this study was provided by the participants’ legal guardians/next of kin. Informed consent was obtained from all individual participants or parents included in the study.

## Author contributions

HT: Conceptualization, Data curation, Investigation, Methodology, Supervision, Visualization, Writing – original draft, Writing – review & editing. ZD: Data curation, Methodology, Writing – review & editing. HS: Writing – review & editing. HD: Methodology, Supervision, Visualization, Writing – review & editing. HX: Conceptualization, Funding acquisition, Methodology, Visualization, Writing – review & editing.
