# Supplementary material for: Single-cell RNA-seq identified novel genes involved in primordial follicle formation
Source: Front Endocrinol (Lausanne). 2023 Dec 11;14:1285667. doi: 10.3389/fendo.2023.1285667 (PMC10750415; doi:10.3389/fendo.2023.1285667)
Supplement: Supplementary file 1 [file DataSheet_1.zip › supplementary materials/Supplementary information.docx]

**Single-cell RNA-seq Identified Novel Genes Involved in Primordial Follicle Formation SUPPLEMENTARY APPENDIX**

Hang-Jing Tan^1 2^, Zi-Heng Deng^1 2^, Hui Shen^3^, Hong-Wen Deng^3^ and Hong-Mei Xiao^1 2^

^1^ Institute of Reproduction and Stem Cell Engineering, School of Basic Medical Science, Central South University, Changsha 410000, China.

^2^ Center for Reproductive Health, and System Biology, Data Sciences, School of Basic Medical Science, Central South University, Changsha 410000, China.

^3^ Center of Biomedical Informatics and Genomics, Deming Department of Medicine, Tulane University School of Medicine, New Orleans, LA, 70112, USA.

**Corresponding Author:**

Dr. Hong-Mei Xiao, Institute of Reproduction and Stem Cell Engineering, School of Basic Medical Science, Central South University, Changsha, 410000, China; hmxiao@csu.edu.cn.

Prof. Hong-Wen Deng, Center of Biomedical Informatics and Genomics, Deming Department of Medicine, Tulane University School of Medicine, New Orleans, LA, 70112, USA; denghongwen66@yahoo.com.

**Table of Contents**

**SUPPLEMENTARY METHODS**

Whole exome sequencing and Sanger sequencing

Enzyme linked immunosorbent assay

**SUPPLEMENTARY FIGURES LEGEND**

**SUPPLEMENTARY METHODS**

**Whole exome sequencing and Sanger sequencing**

Genomic DNA was extracted from peripheral blood for exome sequencing. Exome sequences were captured using the SureSelect Target Enrichment System for Illumina Paired-End Sequencing Library (Agilent Technologies, Santa Clara, CA, USA). DNA sequencing was performed on the Illumina HiSeq Platform (Illumina, San Diego, CA, USA). Reads were mapped to the GRCh37. Variants were annotated using GATK, ANNOVAR, and custom pipelines.

Sanger sequencing were used to confirm the mutation of the candidate genes,*GTF2F1* and *SDC1*, identified via whole exome sequencing. We used specific PCR primers (*GTF2F1*-c.943A>G-F: 5`-CTGCAGAGGTCAGGGTTGG-3` and *GTF2F1*-c.943A>G-R: 5`-GCCCAAGGGTAGGCGATG -3`; *GTF2F1*-c.595C>T-F: 5` Tctcctgcctcagtctccc-3`, and *GTF2F1-*c.595C>T>G-R: 5`-CGGCTCAAGGATCAGGACC -3`; *SDC1*-c.461A>G-F: 5`-CTGTGCCCTGTCTTCCTGG -3` and *SDC1*-c.461A>G-R: 5`-CATCTGGCCTCAACGACCA -3` ) to amplify the target region. The amplified PCR products were run on 2.0% agarose gel electrophoresis. Then, we identified the band size, and the PCR products were sequenced on ABI 3730 automated sequencer (Applied Biosystems, Forster City, CA). PCR-based genotyping of each locus was carried out using the primers designed with Primer5 Software. The PCR reaction conditions was: 95 ℃ initial denaturation 3 min; 38 cycles of 94 ℃ denaturation 40s, 58.5 ℃ annealing 40s, and extension at 72 ℃ for 1 min; 72 ℃ final elongation 5min; 4℃ hold.

**Enzyme linked immunosorbent assay**

GTF2F1 were detected using kits (Jiangsu Meibiao Biological Technology Co.,Ltd). The kit uses a double antibody one-step sandwich enzyme-linked immunosorbent assay (ELISA). Add the sample, standard substance, and HRP labeled detection antibody to the pre coated wells of the universal transcription factor IIF peptide 1 (GTF2F1) antibody, then incubate and thoroughly wash them. Using substrate tetramethylbenzidine (TMB) for color development, TMB is converted into blue under the catalysis of peroxidase, and finally into yellow under the action of acid. The depth of color is positively correlated with the universal GTF2F1 in the sample. Measure the absorbance (OD value) using an enzyme-linked immunosorbent assay at a wavelength of 450nm and calculate the sample concentration.

SDC1 were detected using kits (Jianglai Biological Technology Co.,Ltd). This kit uses a dual antibody sandwich enzyme-linked immunosorbent assay (ELISA). Add samples, standards, biotin labeled detection antibodies, and HRP enzyme conjugates to the micropores pre coated with human multi ligand Syndecan-1 (SDC-1) capture antibodies. The samples are then incubated and washed in the middle, and the substrate TMB is used for color development. TMB is converted into blue under the catalysis of peroxidase (HRP), and finally into yellow under the action of acid. The depth of color is positively correlated with the presence of human multi ligand SDC-1 in the sample. Measure the absorbance (OD value) using an enzyme-linked immunosorbent assay at a wavelength of 450nm and calculate the sample concentration.

**SUPPLEMENTARY FIGURES LEGEND**

**Fig S1** Feature plots of specific marker genes of mice germ cells, granulosa cells, immune cells, stromal cells, erythrocyte cells and endothelial cells.

**Fig S2** Feature plots of specific marker genes of mice germ cells in germline cysts.

**Fig S3** Feature plots of specific marker genes of mice germ cells in primordial follicles.

**Fig S4** (A) Feature plots of specific marker genes of human germ cells. (B-D) Feature plots of specific marker genes of human germ cells in germline cysts and primordial follicles.

**Fig S5** The expression of common TFs in dataset 6 and dataset 7 in different human tissues in the HPA database.
