# Supplementary material for: Single-cell RNA-seq identified novel genes involved in primordial follicle formation
Source: Front Endocrinol (Lausanne). 2023 Dec 11;14:1285667. doi: 10.3389/fendo.2023.1285667 (PMC10750415; doi:10.3389/fendo.2023.1285667)
Supplement: Supplementary file 1 [file DataSheet_1.zip › supplementary materials/Table S1.docx]

| Antibody | Catalog Code | Source | Host | Dilution | |
| --- | --- | --- | --- | --- | --- |
|  |  |  |  | IF | WB |
| DDX4/Ddx4 | 67147-2 | Proteintech | mouse | 1:200 |  |
| MDK/Mdk | BS6038 | Bioworld | rabbit | 1:100 | 1:500 |
| SDC1/Sdc1 | YT5610 | Immunoway | rabbit | 1:100 | 1:1000 |
| Ki67 | YT2467 | Immunoway | rabbit | 1:100 |  |
| ANXA7/Anxa7 | YT5373 | Immunoway | rabbit | 1:100 | 1:1000 |
| GTF2F1/Gtf2f1 | 825097 | Zhengneng | rabbit | 1:100 | 1:1000 |
| Jak2 | YT2428 | Immunoway | rabbit |  | 1:1000 |
| Jak3 | YT2430 | Immunoway | rabbit |  | 1:1000 |
| Stat | YT4443 | Immunoway | rabbit |  | 1:1000 |
| phospho-Stat (Y705) | YP0251 | Immunoway | rabbit |  | 1:1000 |
| Notch2 | YC0069 | Immunoway | rabbit |  | 1:1000 |
| Jagged1 | YT5401 | Immunoway | rabbit |  | 1:1000 |
| Jnk1 | 66210-1 | Proteintech | mouse |  | 1:1000 |
| Jnk2 | YT2442 | Immunoway | rabbit |  | 1:1000 |
| E-cadherin | YT1454 | Immunoway | rabbit |  | 1:1000 |
| Wnt4 | 14371-1 | Proteintech | rabbit |  | 1:1000 |
| PI3K p110γ | BS6441 | Bioworld | rabbit |  | 1:500 |
| phospho-PI3K (Y607) | CBS-PA000712 | Cusabio | rabbit |  | 1:1000 |
| Akt | YP0006 | Immunoway | rabbit |  | 1:1000 |
| phospho-Akt (S473) | YT0178 | Immunoway | rabbit |  | 1:1000 |
| Gapdh | GB15002 | Servicebio | mouse |  | 1:1000 |

**Table S1 Primary antibodies used in the immunodetection**
