# Supplementary material for: Single-cell RNA-seq identified novel genes involved in primordial follicle formation
Source: Front Endocrinol (Lausanne). 2023 Dec 11;14:1285667. doi: 10.3389/fendo.2023.1285667 (PMC10750415; doi:10.3389/fendo.2023.1285667)
Supplement: Supplementary file 1 [file DataSheet_1.zip › supplementary materials/Table S10.docx]

Table S10 Pathogenic variants of *GTF2F1* and *SDC1* identified in POI patients and their clinical characteristics.

| Gene name | Patient number | Variants identified | | Clinical characteristics | | | | | |
| --- | --- | --- | --- | --- | --- | --- | --- | --- | --- |
|  |  | Genotype | Variant | Age at  diagnosis, years | Menarche  onset, years | BMI | AMH,  ng/mL | FSH,  mIU/mL | E2,  pg/mL |
| *GTF2F1* | Patient 1 | Het | c.943A>G | 33 | 13 | 20.3 | <0.01 | 32 | 11.4 |
|  | Patient 2 | Het | c.595C>T | 35 | 14 | 21.5 | 0.04 | 75.19 | 31.48 |
| *SDC1* | Patient 3 | Het | c.461A>G | 30 | 12 | 21.8 | 0.81 | 62.3 | 30.25 |
| AMH antimullerian hormone, BMI body mass index, FSH follicle-stimulating hormone, Het heterozygote, POI premature ovarian insufficiency. | | | | | | | | | |
