# Supplementary material for: Single-cell RNA-seq identified novel genes involved in primordial follicle formation
Source: Front Endocrinol (Lausanne). 2023 Dec 11;14:1285667. doi: 10.3389/fendo.2023.1285667 (PMC10750415; doi:10.3389/fendo.2023.1285667)
Supplement: Supplementary file 1 [file DataSheet_1.zip › supplementary materials/Table S11.docx]

Table S11 *In silico* analysis of GTF2F1 and SDC1 variants

| Gene name | Patient number | Variant identified | In silico tools | | | | | | | | | |  |  |
| --- | --- | --- | --- | --- | --- | --- | --- | --- | --- | --- | --- | --- | --- | --- |
|  |  |  | Mutation Taster | CADD | DANN | FATHMM_MKL | fitCons | GERP++ | phyloP | phastCons | SIFT | POLYPHEN | PROVEAN | GenoCanyon |
| GTF2F1 | Patient 1 | c.943A>G | Disease_causing | Damaging | Damaging | Damaging | Damaging | Conserved | Conserved | Conserved | Tolerable | Benign | Tolerable | Tolerable |
|  | Patient 2 | c.595C>T | Disease_causing | Damaging | Damaging | Damaging | Damaging | Conserved | Nonconserved | Nonconserved | Damaging | Benign | Damaging | Tolerable |
| SDC1 | Patient 3 | c.461A>G | Polymorphism | Tolerable | Tolerable | Tolerable | Damaging | Nonconserved | Nonconserved | Nonconserved | Tolerable | Probably_damaging | Tolerable | Damaging |
