# Supplementary material for: Single-cell RNA-seq identified novel genes involved in primordial follicle formation
Source: Front Endocrinol (Lausanne). 2023 Dec 11;14:1285667. doi: 10.3389/fendo.2023.1285667 (PMC10750415; doi:10.3389/fendo.2023.1285667)
Supplement: Supplementary file 1 [file DataSheet_1.zip › supplementary materials/Table S2.docx]

| Gene | Sequence |
| --- | --- |
| sh-Anxa7 | GCTGAACGACTCTACTATT |
| sh-Gtf2f1 | AGCGAGATCTGAGCAACAA |
| sh-Mdk | GAGTGTTCGGAGTGGACCT |

Table S2 Sequence used for shRNA lentivirus and siRNA production
