# Supplementary figures and images for: Single-cell RNA-seq identified novel genes involved in primordial follicle formation
Source: Front Endocrinol (Lausanne). 2023 Dec 11;14:1285667. doi: 10.3389/fendo.2023.1285667 (PMC10750415; doi:10.3389/fendo.2023.1285667)

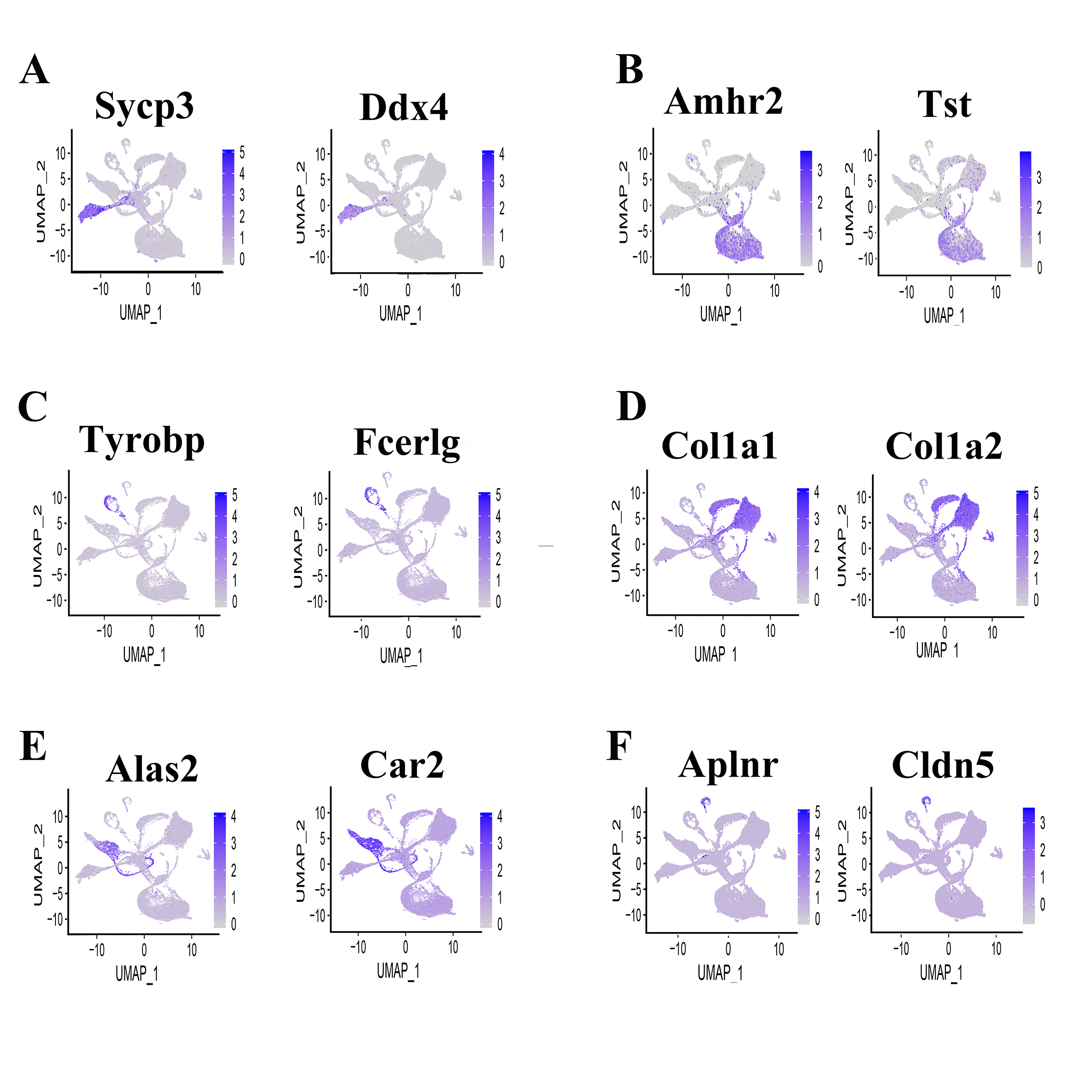

Supplement: Supplementary file 1 [file DataSheet_1.zip › supplementary materials/Fig S1.tif]

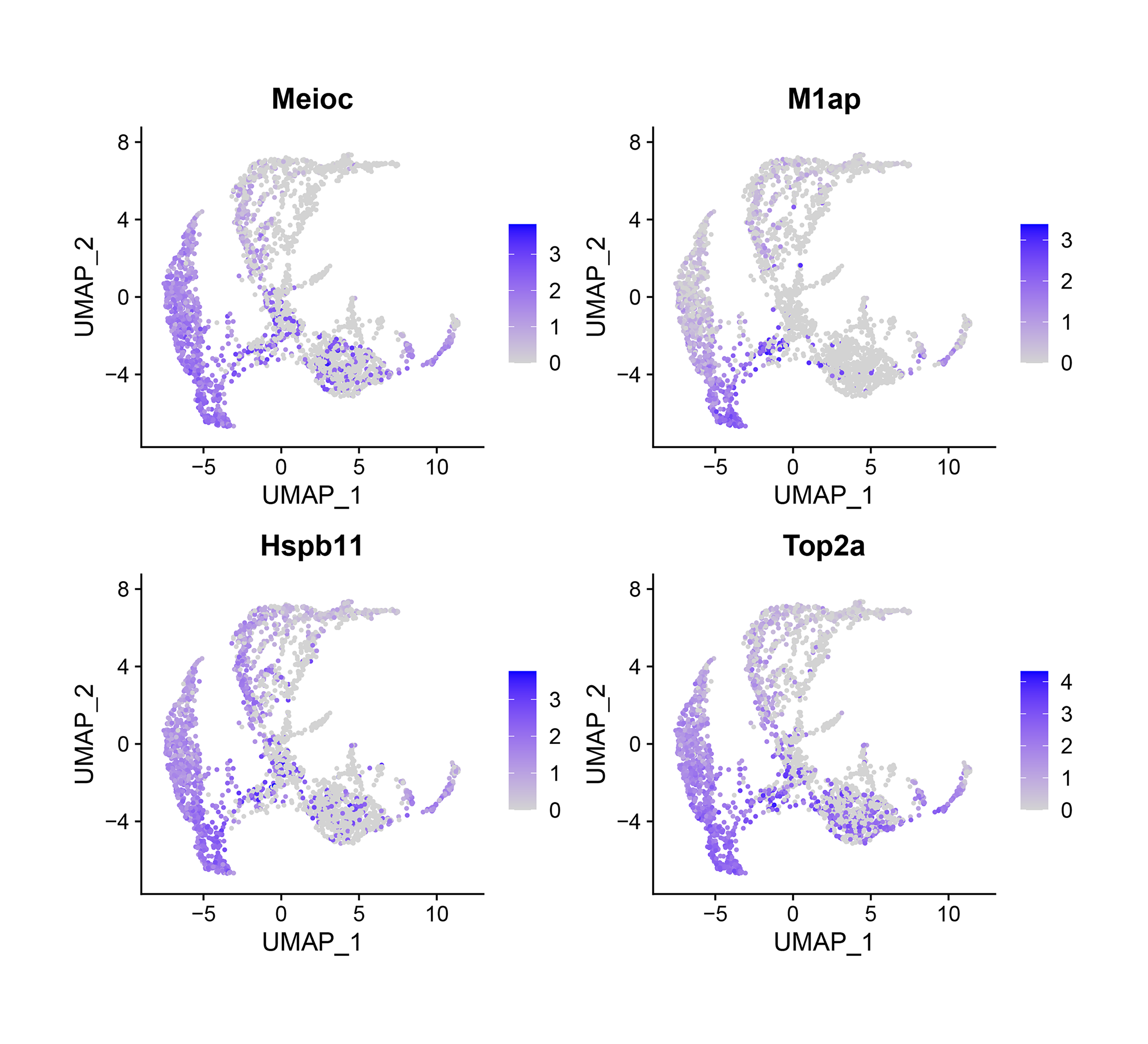

Supplement: Supplementary file 1 [file DataSheet_1.zip › supplementary materials/Fig S2.tif]

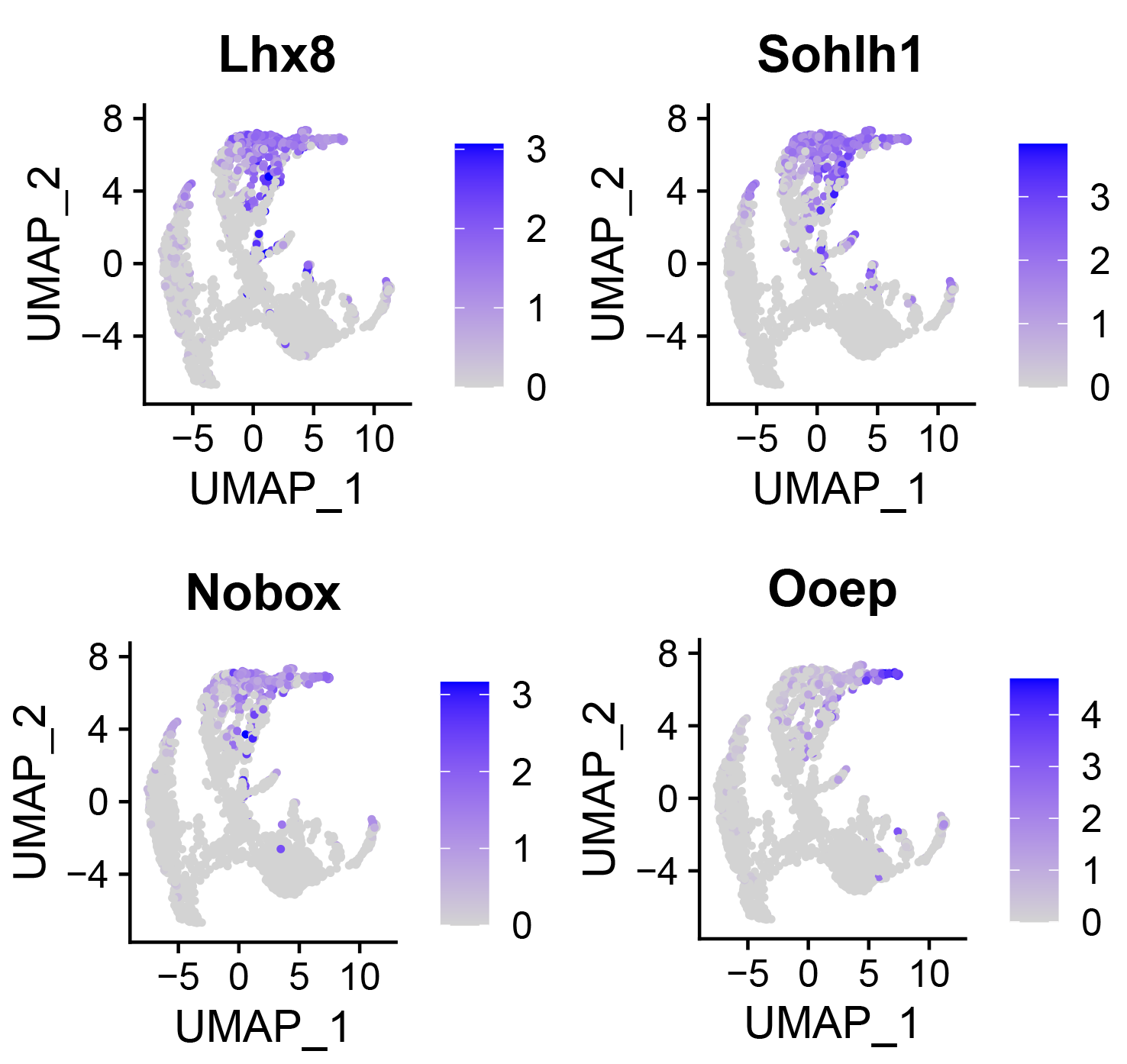

Supplement: Supplementary file 1 [file DataSheet_1.zip › supplementary materials/Fig S3.tif]

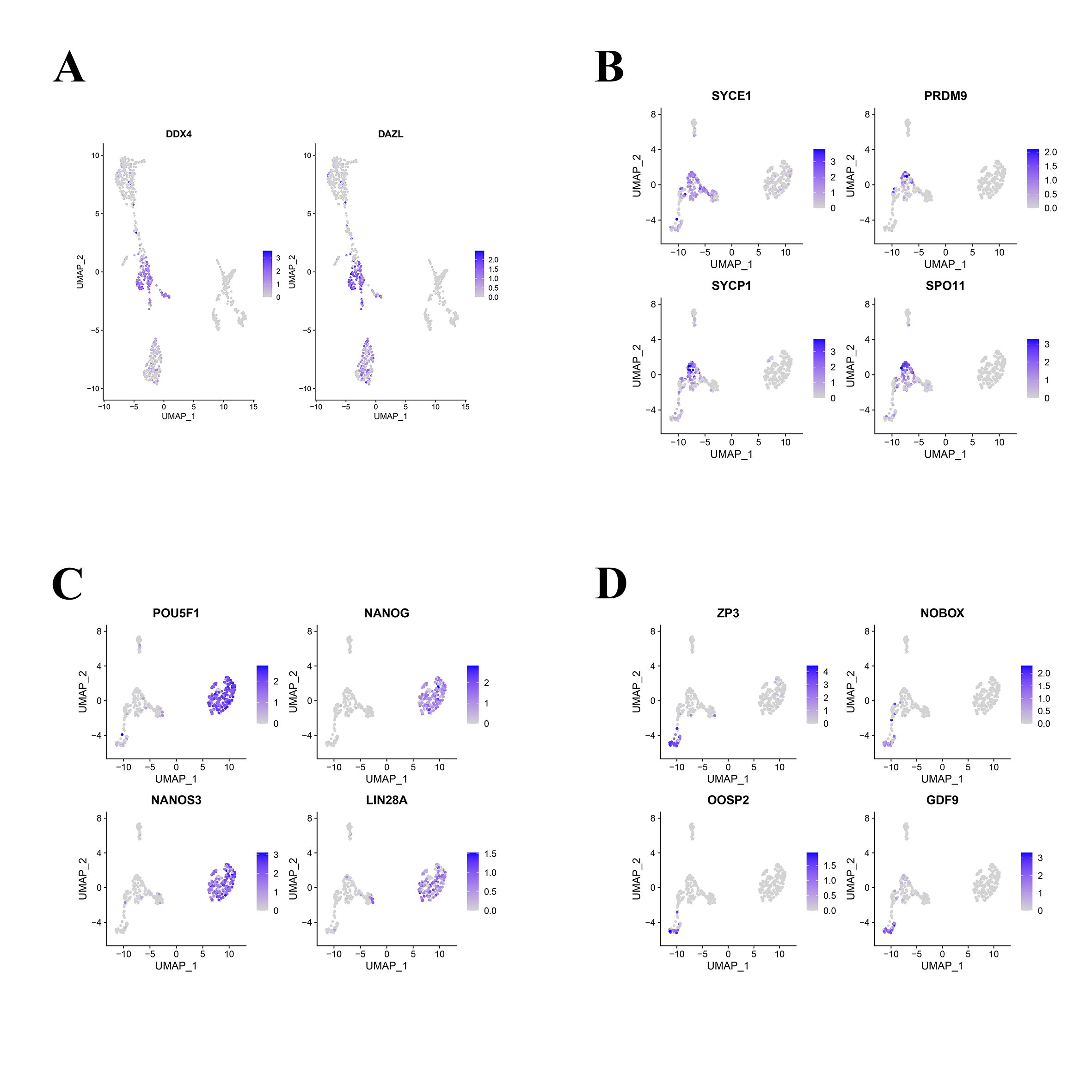

Supplement: Supplementary file 1 [file DataSheet_1.zip › supplementary materials/Fig S4.tif]

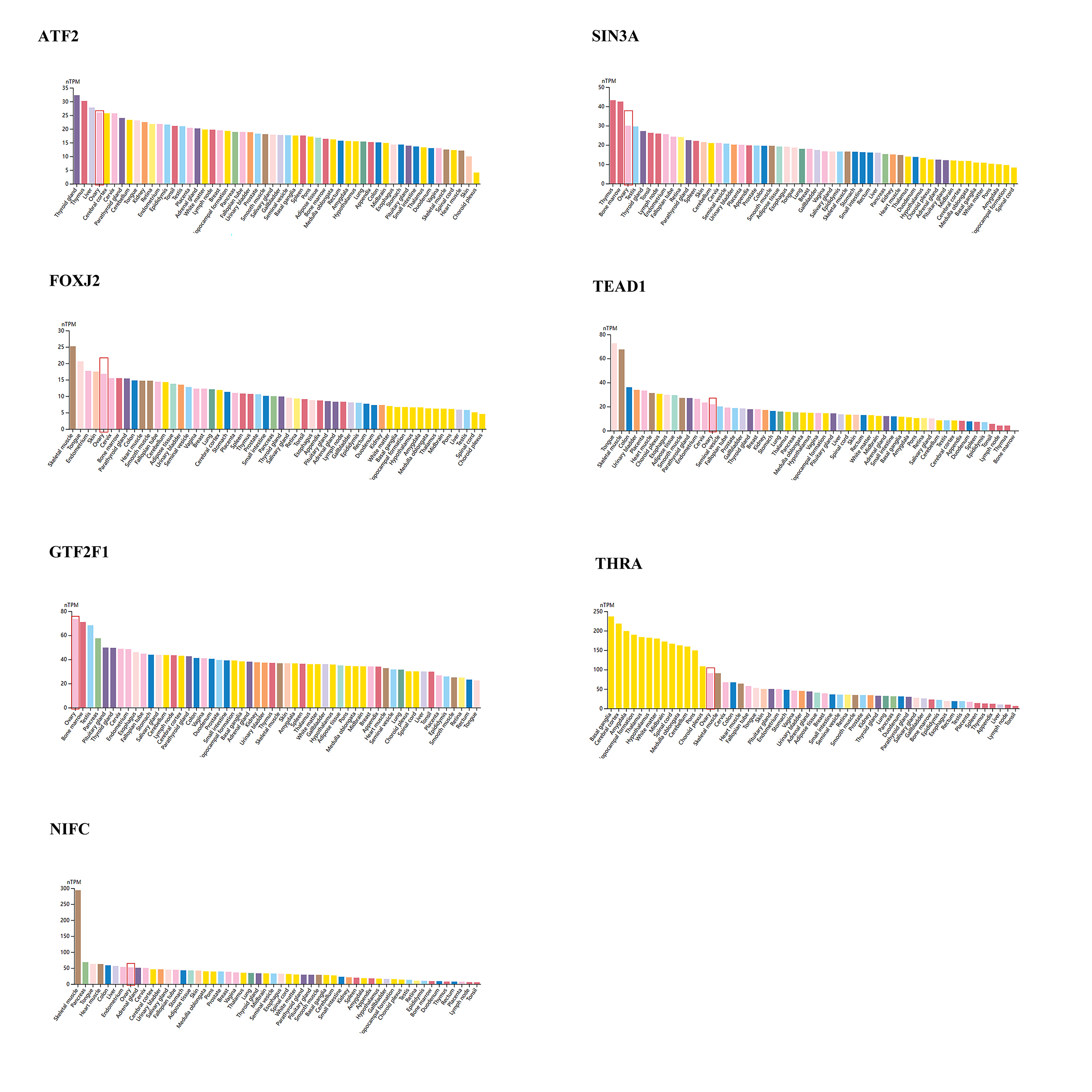

Supplement: Supplementary file 1 [file DataSheet_1.zip › supplementary materials/Fig S5.tif]
